# Gender differences for frailty in HIV-infected patients on stable antiretroviral therapy and with an undetectable viral load

**DOI:** 10.1371/journal.pone.0215764

**Published:** 2019-05-09

**Authors:** José-Ramón Blanco, Inmaculada Barrio, Enrique Ramalle-Gómara, María Isabel Beltran, Valvanera Ibarra, Luis Metola, Mercedes Sanz, José A. Oteo, Estrella Melús, Lucía Antón

**Affiliations:** 1 Departamento de Enfermedades Infecciosas, Hospital Universitario San Pedro & Centro de Investigación Biomédica de La Rioja (CIBIR), Logroño, Spain; 2 Departamento de Enfermedades Infecciosas, Hospital Universitario San Pedro, Logroño, Spain; 3 Consejería de Salud de La Rioja, Gobierno de La Rioja,Logroño, Spain; 4 Escuela Universitaria de Enfermería de La Rioja, Logroño, Spain; Institut Hospital del Mar d'Investigacions Mediques, SPAIN

## Abstract

**Background:**

Patients with HIV infection suffer from accelerated aging. In this context, frailty could be a relevant problem that aggravates the quality of life (QoL) and morbi-mortality of these patients. Our objective was to determine the prevalence of frailty and pre-frailty in HIV-infected patients in our cohort as well as their risk factors and QoL.

**Methods:**

This was a prospective cross-sectional study of HIV-infected people aged ≥18 years on a stable antiretroviral regimen (ART) ≥1 year. Frailty was defined by ≥3 of 5 Fried's criteria: weight loss, low physical activity, exhaustion, weak grip strength and slow walking time. Variables related to sociodemographics, HIV infection, comorbidities, polypharmacy, and QoL were evaluated. Independent predictors of frailty were evaluated using collinearity in a multivariate logistic regression analyses (backward stepwise elimination).

**Results:**

The 248 people studied has a mean age of 49 years, 63.7% were male, and 81% were Caucasian. The prevalence of pre-frailty and fragility was 39.1% and 4.4%, respectively. The main route of HIV acquisition was heterosexual (47.2%). At the inclusion time 26.6% of the patients had AIDS events, 60.9% were anti-HCV negative, and 91.5% had HIV RNA <50 copies/mL (84.3% for ≥1 year); 10.9% had >2 comorbidities, and 13.3% were receiving >5 non-HIV drugs. Frailty patients had a higher age (p 0.006), more sensitive deficits (visual or auditory) (p 0.002), a greater number of falls during the previous year (p 0.0001), a higher Charlson comorbidity index (p 0.001), and a higher VACS index (p 0.001). All comorbidities, excluding bone and liver, were significantly more frequent in fragile patients. The presence of >2 comorbidities and treatment with >5 drugs not related to HIV they were also more frequent in frail patienst (p 0.0001 and p 0.004, respectively). Independent predictors of pre-frailty/frailty in the multivariable analysis differ in men (VACS index, C-reactive protein [CRP], and falls) and women (CRP, AIDS, and menopause). Patients with pre-frailty/frailty had some indicator of a lower QoL.

**Conclusion:**

Factors associated with pre-frailty/frailty in HIV-infected patients differ by gender, which should be considered when establishing measures for prevention. The role of menopause in the risk of pre-frailty/frailty warrants further investigations.

## Introduction

Effective treatments have transformed HIV infection into a chronic disease [1]. As a result, HIV-infected individuals are living longer. In 2014, the Joint United Nations Program on HIV/AIDS (UNAIDS) estimated that approximately 4.2 million people aged 50 years or more worldwide are living with HIV [2]. This success has led to the progressive aging of these patients and, as a consequence, the emergence of multiple age-related diseases.

Although aging affects all people, not everyone ages in the same way. This variable risk of poor health in people of the same age is known as frailty [3]. Frailty is defined as a state of increased vulnerability to the poor resolution of homeostasis following a stress, which increases the risk of adverse outcomes including falls, delirium and disability [4, 5]. Frailty is an important complex condition of public health interest that is now routinely observed among HIV-infected patients. In this population, the prevalence of frailty varies from 5–28.6% depending on the population studied [6]. This prevalence is not only higher than in HIV-uninfected people but it also appears in younger ages [7].

The objective of this study was to examine the current prevalence of frailty using Fried et al.’s criteria and identify the factors that may increase its risk in HIV-infected patients.

## Materials and methods

### Ethics statement

This study was approved by the Institutional Research Ethics Committee (Comité de Ética de Investigación con medicamentos de La Rioja [CEImLAR]). All participants provided written informed consent.

### Participants

This was a prospective cross-sectional study of HIV-infected patients aged >18 years on stable ART for at least 12 months who provided consent. Pregnant women and breast-feeding mothers, patients employing illicit drugs other than marijuana, alcohol abuse or dependence, a history of active cancer in the prior 12 months and treatment for this disease during the same period of time or HCV with on treatment during the prior 6 months were also excluded.

The socio-demographic information and medical history were obtained via a questionnaire. Education status, residence, marital status, smoking status and alcohol use were determined from self-administered survey responses. Anthropometric measurements included body mass index (BMI), and waist/hip ratio. Physical examination was also performed to evaluate any changes in lipodystrophy. This included a specific assessment of lipoatrophy and lipohypertrophy of any severity [8].

Non-AIDS events (NAEs) data records included in this study were: (i) cardiovascular disease (coronary heart diseases, cerebrovascular diseases, peripheral arterial diseases, chronic heart failure), (ii) hepatic diseases (cirrhosis), (iii) metabolic and endocrine disorders (diabetes mellitus, metabolic syndrome), (iv) renal diseases (chronic renal failure), (v) lung disease (chronic obstructive pulmonary diseases), (vi) neuropsychiatric diseases (depression, dementia, schizophrenia, bipolar disorder, psychosis), and (vii) bone disease (osteoporosis, bone fracture). Falls or previous hospital admission in the previous year and hearing or visual problemas were also evaluated.

Multimorbidity was defined as the presence of >2 NAEs and polypharmacy as the use of >5 chronic non-ART drugs. The Charlson comorbidity index [9] was used to quantify comorbid disease burden. Another mortality index, the VACS index [10], was also employed. Analytical parameters commonly employed in daily clinical practice were also evaluated. In women, specific questions about previous pregnancy, number of biths and menopause were also evaluated. Menopause was defined as 12 consecutive months without menstruation [11].

HIV-related characterisitics included the route of acquisition, previous AIDS events, years of HIV infection, current CD4-T cell count (cell/mm^3^ and percentage), CD8-T cell count (cell/mm^3^ and percentage), CD4/CD8 ratio, CD4-T cell nadir, HIV viral load (current and manteined during at least one year), ART regimen, years of ART, and ART adherence. HCV coinfection, chronic HbsAg and FIB-4 [12] were also evaluated.

Patients’ overall quality of life (QoL) was assessed using the Medical Outcomes Study HIV Health Survey (MOS-HIV) and EQ-5D questionnaires [13]. The MOS-HIV is a 35-item, self-administered questionnaire that consists of eight multi-item subscales (health perceptions, physical function, role function, cognitive function, pain, mental health, energy/fatigue, and health distress) and two single-item subscales (social function, quality of life) [14]. The scales of the MOS-HIV are scored as summated rating scales on a 0–100 scale, where higher scores indicate a better health status. In addition to these subscales, a physical health summary score (PHS) and a mental health summary score (MHS) were calculated by standardizing the scores in these domains using weighting coefficients. The PHS is weighted toward the pain, health perceptions, physical, role, and social function scales [15, 16]. The MHS strongly reflects the mental health, health distress, overall quality of life, cognitive function, and energy/fatigue scales [15, 16].

The EQ-5D is a five-item instrument with one question assessing each of five dimensions: mobility, self-care, usual activities, pain/discomfort, and anxiety/depression [17]. Higher scores indicate better health. The EQ-5D also includes a visual analog scale (EQ-5D VAS) that assesses overall health. VAS scores range from 0 to 100 and higher scores indicate better health. Both MOS-HIV and EQ-50 instruments are widely used in clinical trials and observational studies [13]. Indeed, these two instruments may complement each other [13].

Finally, a self-related health-perception was also performed. The responses were ranked from 1 to 4 (excellent  =  1; good  =  2; fair  =  3; bad  =  4). For the present analyses, these responses were dichotomized into two categories, good or excellent  =  1 and poor or fair  =  0.

### Frail phenotype

The frailty status assessment was based on the 5-item criteria described by Fried et al. [4]. Briefly, participants were scored on a 0–1 scale (1 being at risk and 0 being not at risk for frailty) for 5 components: weakness (grip strength), slowness (time to walk 4.57 m), exhaustion (self-reported), low physical activity level (measured through a weighted score of kilocalories expended per week), and weight loss (self-reported). According to this score, the patients were classified as non-frail or robust (0 component), pre-frail (1 or 2 components), or frail (≥3 components). Grip strength of the dominant hand was measured three times using a grip dynamometer (Jamar Plus+ Digital Hand Dynamometer, Jamar, US). The average of three weight measurements was recorded in kilograms (kg) to one decimal point. Walk distance at the patient’s own usual pace was calculated according to the average of two consecutive measurements. Participants were excluded from the determination of grip strength if they had pain or arthritis of the dominant hand, and excluded from the gait speed analysis if they had paralysis of an extremity or side of the body, or needed to use a walking aid [18].

### Statistical analysis

Descriptive statistics were employed to describe all the quantitative variables. Characteristics were expressed as the mean ± standard deviation (SD) or the percentage. We used the chi-square test for comparisons of qualitative data, analysis of variance (ANOVA) for continuous variables, and the non-parametric test when necessary. After testing the proportionality of odds across the robust, pre-frail and frail categories using a likelihood ratio test, a multivariate logistic regression analyses (backward stepwise elimination) was constructed. We entered into the model variables that were associated in the bivariate analysis with a p-value <0.20, excluding variables that presented collinearity with other factors. Statistical significance was set at p <0.05 and statistical analyses were performed using SPSS 24.0 software (SPSS Inc., Chicago, IL, USA).

## Results

Frailty assessment was performed in 248 HIV-infected patients. The characteristics of the patients are shown in Table 1. The prevalence of frailty and pre-frailty was 4.4% and 39.1%, respectively. Frailty was associated with a mean older age (59.5 years vs. 47.7 years in robust) (p 0.006), and feeling older than actual age (p 0.03). Groups were comparable in terms of gender, race, education rate, urban place of residence, marital status, tobacco, alcohol and marijuana use. In women, no differences were observed after analysis of previous pregnancy, number of births or menopause.

**Table 1:** Characteristics of the patients according to frailty status.

	Robust (n = 140)	Prefrail (n = 97)	Frail (n = 11)	*P* value
Age in years, mean (± SD)	47.7 (8.6)	49.6 (11.6)	59.5 (12.7)	0.006 ^a^
>50 years, n (%)	66 (47.1)	57 (58.8)	8 (72.7)	0.08
Feeling older than actual age, n (%)	18 (12.9)	18 (18.6)	5 (45.5)	0.03
Male sex, n (%)	97 (69.3)	55 (56.7)	6 (54.5)	0.11
Race White, n (%)	111 (79.3)	81 (83.5)	9 (81.8)	0.9
Educational level, n (%) No school attendance Primary school Secondary school Higher education	11 (7.9) 49 (35.0) 61 (43.6) 19 (13.6)	6 (6.2) 47 (48.5) 35 (36.1) 9 (9.3)	2 (18.2) 5 (45.5) 3 (27.3) 1 (9.1)	0.30
Urban place of residence, n (%)	118 (84.3)	81 (83.5)	10 (90.9)	0.79
Marital status, n (%) Single Married Living with a partner Separate Widow/Widower	57 (40.7) 57 (40.7) 4 (2.9) 16 (11.4) 6 (4.3)	39 (40.2) 35 (36.1) 3 (3.1) 15 (15.5) 5 (5.2)	5 (45.5) 4 (36.4) 1 (9.1) 1 (9.1) 0	0.89
Live, n (%) Alone Husband/wife Friends	40 (28.6) 97 (69.3) 3 (2.1)	29 (29.9) 67 (69.1) 1 (1.0)	5 (45.5) 6 (54.5) 0	0.69
Smoker status, n (%) Never smoker Former smoker Current smoker	45 (32.1) 35 (25.0) 60 (42.9)	25 (25.8) 18 (18.6) 54 (55.7)	2 (18.2) 2 (18.2) 7 (63.6)	0.29
Marijuana smoker, n (%)	20 (14.3)	12 (12.4)	3 (27.3)	0.46
Alcohol consumption, n (%) Abstemious Low consumption Moderate consumption High consumption	53 (37.9) 52 (37.1) 31 (22.9) 4 (2.9)	36 (37.1) 28 (28.9) 31 (32.0) 2 (2.1)	4 (36.4) 5 (45.5) 2 (18.2) 0	0.62
Previous pregnancy, n (%)	32 (74.4)	30 (71.4)	4 (80.0)	0.92
Number of birth, mean (± SD)	1.4 (1.1)	1.5 (1.7)	1.2 (0.8)	0.90
Menopause, n (%)	10 (23.3)	18 (42.9)	2 (40.0)	0.14
Body mass index (kg/m^2^), media (± SD)	24.8 (3.8)	25.1 (4.4)	23.6 (5.5)	0.42
Waist/hip ratio, media (± SD)	0.9 (0.1)	0.9 (0.1)	0.9 (0.1)	0.07
Lipoatrophy, n (%)	11 (7.9)	13 (13.4)	2 (18.2)	0.28
Lipoaccumulation, n (%)	9 (6.4)	9 (9.3)	4 (36.4)	0.025
Hearing or visual problems, n (%)	13 (9.3)	14 (14.4)	6 (54.5)	0.002
Hospital admission in the last year, n (%)	7 (5.0)	5 (5.2)	2 (18.2)	0.32
Falls in the last year, n (%)	4 (2.9)	8 (8.2)	5 (45.5)	0.0001
Charlson index, mean (± SD)	1.9 (2.9)	1.9 (3.0)	5.3 (3.1)	0.001 ^a,b^
VACS index score, mean (± SD)	13.2 (11.1)	17.7 (11.2)	24.7 (14.3)	0.001 ^a,c^

**Note**: FIB-4: Fibrosis-4 index; SD = Standard deviation.

P-value is significant at the 0.05 level (^a^ robust vs frail; ^b^ prefrail vs frail; ^c^ robust vs prefrail).

In the physical examination, no differences in BMI and waist/hip ratio were observed. Lipoaccumulation was more common in frailty people (p 0.025).

Overall, the most common comorbid conditions were cardiovascular (27%), neurologic (25.4%), endocrine (25%), lung (8.9%), and renal (4.8%). All of them were more common in frail people (all p <0.02). No differences were observed in liver and bone conditions. Frail people also showed a higher prevalence of more than 2 comorbid conditions (63.6%) and polypharmacy (54.5%). The Charlson index and VACS index score were more prevalent in frail people (both p 0.001 in comparisons of robust vs frail and pre-frail vs frail). Frail people also had more hearing or visual problems (54.5%; p 0.002) and falls in the previous years (45.5%; p 0.0001). These findings are shown in Table 2.

**Table 2:** Comorbidities and polypharmacy.

	Robust	Prefrail	Frail	*P* value
Cardiovascular comorbid conditions, n (%)	33 (23.6)	26 (26.8)	8 (72.7)	0.004
Liver comorbid conditions, n (%)	7 (5.0)	4 (4.1)	1 (9.1)	0.63
Endocrine comorbid conditions, n (%)	28 (20.0)	27 (27.8)	7 (63.3)	0.008
Lung comorbid conditions, n (%)	14 (10.0)	4 (4.1)	4 (36.4)	0.007
Bone comorbid conditions, n (%)	6 (4.3)	5 (5.2)	2 (18.2)	0.27
Neurologic comorbid conditions, n (%)	28 (20.0)	29 (29.9)	6 (54.5)	0.02
Renal comorbid conditions, n (%)	5 (3.6)	3 (3.1)	4 (36.4)	0.001
Comorbid conditions >2, n (%)	14 (10.0)	6 (6.2)	7 (63.6)	0.0001
Polypharmacy, n (%)	16 (11.4)	11 (11.3)	6 (54.5)	0.004

Table 3 shows the analytical parameters. Frail people had significantly higher mean creatinine levels (p 0.03) and lower estimated glomerular filtration rates (p 0.004 in comparisons of robust vs frail and pre-frail vs frail). Similarly, ALT and albumin were also lower in frail people (p 0.02 and p 0.001, respectively). Moreover, CRP was higher in frail people (p 0.01 in comparisons of robust vs pre-frail). No differences were observed after analyzing the other parameters.

**Table 3:** Analytical parameters.

	Robust	Prefrail	Frail	*P* value
Hemoglobin, g/dL, mean (± SD)	14.5 (1.4)	14.1 (1.5)	14.2 (1.4)	0.12
Hematocrit %, mean (± SD)	44.0 (4.3)	42.9 (4.3)	43.2 (4.5)	0.14
Platelets (x10^3^/uL), mean (± SD)	213.3 (56.3)	214.6 (63.4)	212.2 (59.2)	0.95
Glucose mg/dL, mean (± SD)	93.9 (16.2)	94.4 (19.2)	102.0 (17.4)	0.10
Creatinine mg/dL, mean (± SD)	0.9 (0.1)	0.8 (0.2)	1.3 (1.1)	0.03
Estimated glomerular filtration rate (ml/min/1.73 m^2^) (n, %)	92.6 (16.5)	92.2 (18.7)	66.9 (26.5)	0.004 ^a,b^
HDL-c, mg/dL mean (± SD)	50.6 (14.4)	52.0 (16.4)	52.6 (18.7)	0.64
LDL-c, mg/dL, mean (± SD)	101.5 (27.0)	106.9 (32.4)	114.9 (35.0)	0.43
Total cholesterol, mg/dL, (mean (± SD)	178.9 (33.1)	184.3 (36.8)	194.9 (38.3)	0.54
Triglycerides, mg/dL, mean (± SD)	146.2 (122.2)	136.8 (79.2)	150.1 (105.5)	0.96
AST, IU/L, mean (± SD)	23.2 (9.9)	22.3 (12.3)	19.5 (7.4)	0.09
ALT, IU/L, mean (± SD)	25.3 (13.9)	23.3 (17.6)	18.6 (8.2)	0.02
Total bilirubin, mean (± SD)	0.7 (0.9)	0.6 (0.5)	0.3 (0.1)	0.07
Albumin, g/dL, mean (± SD)	4.5 (0.3)	4.5 (0.2)	4.2 (0.8)	0.001 ^a,b^
Total proteins, g/dL, mean (± SD)	7.2 (0.4)	7.2 (0.4)	6.8 (0.4)	0.06
C-reactive protein g/dL, mean (± SD)	2.0 (2.4)	5.3 (18.0)	21.8 (53.5)	0.01 ^c^
Fibrinogen, mg/dL, mean (± SD)	356.4 (62.7)	371.5 (84.5)	410.7 (114.0)	0.13

**Note**: ALT: Alanine amino transferase; AP: Alkaline phosphatase; AST: Aspartate amino transferase; HDL-c: High-density lipoprotein cholesterol; FIB-4: Fibrosis-4 index; GGT: Gamma-glutamyl transferase; LDL: Low-density lipoprotein cholesterol; SD = Standard deviation; TC: Total cholesterol; TGD: Triglycerides. P-value is significant at the 0.05 level (^a^ robust vs frail; ^b^ prefrail vs frail; ^c^ robust vs prefrail).

HIV disease characteristics according to the frailty phenotype are shown in Table 4, demonstrating that the details were comparable between the groups for all the analyzed variables.

**Table 4:** HIV and ART-related characteristics of HIV-infected participants.

	Robust	Prefrail	Frail	*P* value
HIV route of acquisition, n, (%) Injecting drug use	48 (34.3)	37 (38.1)	5 (45.5)	0.20
Previous AIDS events, n (%)	38 (27.1)	23 (23.7)	5 (45.5)	0.32
Duration of HIV infection (years), mean (± SD)	14.7 (8.3)	15.6 (8.5)	16.9 (7.8)	0.51
Duration of ART (years), mean (± SD)	12.7 (7.5)	12.9 (7.5)	15.7 (6.9)	0.42
Current or previous exposure to NRTI, n (%)	137 (97.9)	97 (100)	11 (100)	0.07
Current or previous exposure to NNRTI, n (%)	93 (66.4)	65 (67.0)	10 (90.9)	0.17
Current or previous exposure to PIs, n (%)	92 (65.7)	64 (66.0)	9 (81.9)	0.51
Current or previous exposure to INSTI, n (%)	34 (24.3)	27 (27.8)	3 (27.3)	0.82
Current number of ART, mean (± SD)	2.3 (1.3)	2.4 (1.5)	2.3 (1.5)	0.96
ART adherence, n (%) <85% 85–99% 100%	4 (2.9) 131 (93.6) 5 (3.6)	4 (4.1) 86 (88.7) 7 (7.2)	1 (9.1) 10 (90.9) 0	0.39
Current CD4+ T-cell/mm^3^, mean (± SD)	789.9 (353.6)	788.4 (360.4)	759.8 (464.7)	0.97
Current CD4%, mean (± SD)	34.8 (10.7)	35.6 (10.7)	29.5 (11.98	0.27
Current CD8+ T-cell/mm^3^, mean (± SD)	918.0 (474.4)	864.8 (385.2)	1008.8 (419.4)	0.47
Current CD8%, mean (± SD)	39.3 (11.0)	39.6 (12.1)	44.2 (10.3)	0.24
Current CD4/CD8 ratio, mean (± SD)	1 (0.5)	1 (0.5)	0.7 (0.4)	0.14
Nadir CD4+ T-cells/mm^3^, mean (± SD)	304.6 (228.8)	329.3 (254.9)	218.1 (183.1)	0.36
Current HIV-RNA <50 cop/mL, n (%)	130 (92.9)	86 (88.7)	11 (100)	0.20
HIV-RNA <50 cop/mL >1 year, n (%)	121 (86.4)	78 (80.4)	10 (90.9)	0.37
HCV coinfection, n (%) Never Cleared Active	86 (61.4) 50 (35.7) 4 (2.9)	58 (59.8) 36 (37.1) 3 (3.1)	7 (63.6) 4 (36.4) 0	0.99
Chronic HBsAg positive, n (%)	5 (3.6)	4 (4.1)	0	1
FIB-4, mean (± SD)	1.1 (0.6)	1.2 (0.7)	1.4 (0.7)	0.58

**Note:** INSTI = Integrase strand transfer inhibitors (INSTIs); NNRTI = Non-nucleoside reverse transcriptase inhibitors; NRTI = Nucleoside reverse transcriptase inhibitors, PIs = Protease inhibitors; SD = Standard deviation. P-value is significant at the 0.05 level (^a^ robust vs frail; ^b^ prefrail vs frail; ^c^ robust vs prefrail).

Because of the low prevalence of frailty, we combined the pre-frail and frail states to increase the statistical power according to Escota et al. [19]. The multivariable analysis showed that factors associated with pre-frailty/frailty differ by gender. For men they were falls in the last year (OR 4.43; CI 95% 1.07–18.37; p 0.04), CRP (OR 2.89; CI 95% 1.24–6.74; p 0.014), and VACS index (OR 2.25; CI 95% 1.13–4.50; p 0,021). CRP (OR 9.29; CI 95% 2.0–43.07; p 0.004); AIDS (OR 3.94; CI 95% 1.21–12.82; p 0.023), and menopause (OR 3.53; CI 95% 1.28–9.67; p 0.014) in women. Table 5 shows a summary of the results.

**Table 5:** Multivariable analysis stratified by sex.

	Crude OR	P (95% CI)	Adjusted OR^a^	P (95% CI)
**Men**				
Age >50 years	8.59	<0.01 (1.38–5.47)		
Falls in the last year	5.80	<0.02 (1,20–18.59)	**4.43**	**0.04 (1.07–18.37)**
Hearing or visual problems	6.75	<0.01 (1.29–8.46)		
Lypoatrophy	3.39	0.06 (0.92–6.48)		
Undetectable HIV viral load	2.13	0.14 (0.12–1.38)		
CKD-EPI <60	3.17	0.07 (0.07–1.21)		
Charlson index (≥2) ^1^	1.86	0.17 (0.81–3.14)		
VACS index (≥15) ^1^	9.18	0.002 (1.41–5.34)	**2.25**	**0.021 (1.13–4.50)**
C-reactive protein (≥4 g/dL) ^2^	8.37	0.004 (1.42–7.21)	**2.89**	**0.014 (1.24–6.74)**
Current smoker	3.70	0.05 (0.98–3.60)		
AIDS events	1.72	0.18 (0.79–3,21)		
Waist/hip ratio (<0.9) ^1^	1.66	0.19 (0.78–3.21)		
Total Protein (<7 g/dL) ^1^	2.01	0.15 (0.32–1,19)		
FIB-4 (≥1.3) ^2^	2.67	0.10 (0.89–3.27)		
Cardiovascular comorbid conditions	2.14	0.14 (0.83–3.42)		
Endocrine comorbid conditions	2.60	0.10 (0.88–3.64)		
Neurologic comorbid condition	4.66	0.03 (1.06–4.90)		
Renal comorbid conditions	2.02	0.15 (0.64–12.15)		
**Women**				
Menopause	3.76	0.052 (0.98–6.09)	**3,53**	**0.014 (1.28–9.67)**
AIDS events	3.91	0.048 (0.13–1.01)	**3.94**	**0.023 (1.21–12.82)**
Falls in the last year	2.49	0.11 (0.56–44.64)		
Place of residence (urban)	1.81	0.17 (0.68–7.25)		
Injecting drug use	1.78	0.18 (0.21–1.34)		
C-reactive protein (>4 g/dL) ^2^	5.56	0.018 (1.19–17.52)	**9.29**	**0.004 (2.0–43.07)**
Endocrine comorbid conditions	2.53	0.11 (0.80–6.89)		

**Note:** FIB-4: Fibrosis-4 index.

^a^ Adjusted for all variables listed including collinearity between variables.

^1^ Below media vs above media

^2^ Above media vs below media

Finally, different scores for QoL were included in this study. Table 6 shows a summary of the EQ-5D, MOS-HIV and self-related health scores. In men, pre-frail/frail patients showed a significant negative effect on QoL based on the EQ-5D and EQ-5D VAS (p <0.0001 and p<0.005, respectively). Similarly, these patients also reported a poorer self-reported health status (p <0.02). Finally, MOS-HIV showed that pre-frail/frail people had a poorer PHS (p <0.003) but no differences were observed after analyzing mental health status. In women, pre-frail/frail patients also showed a significant negative effect on QoL based on the EQ-5D (p <0.04) and MOS-HIV (PHS) (p <0.004). In multivariable analysis, and excluding variables that presented collinearity with other factors, there was only a significant association between pre-frailty/frailty and EQ-5D for men (<0.0001) and MOS-HIV (PHS) for women (<0.004).

**Table 6:** Summary of the questionnaires evaluating the quality of life based on the sex of the patients.

	Robust	Pre-frail/Frail	*P* value
**Men**			
EQ-5D, mean (± SD)	0.94 (0.12)	0.79 (0.25)	**<0.0001** ^a^
EQ-5D visual analog scale, mean (± SD)	76.3 (14.5)	68.8 (18.1)	<0.005
MOS-HIV, Physical health summary score, mean (± SD)	47.3 (4.5)	44.8 (6.2)	0.003
MOS-HIV, Mental health summary score, mean (± SD)	46.9 (7.6)	47.0 (10.3)	0.9
Self rated health Very good/good, n (%)	87 (89.6)	46 (75.4)	<0.02
**Women**			
EQ-5D, mean (± SD)	0.91 (0.14)	0.82 (0.25)	<0.04
EQ-5D visual analog scale, mean (± SD)	78.9 (17.8)	73.7 (22.0)	0.2
MOS-HIV, Physical health summary score, mean (± SD)	47.6 (3.5)	42.2 (6.8)	**<0.004** ^a^
MOS-HIV, Mental health summary score, mean (± SD)	46.1 (12.5)	47.3 (9.9)	0.6
Self rated health Very good/good, n (%)	35 (81.3)	35 (74.4)	0.4

**Note**. EQ-5D = EuroQol five-dimensional; EQ-5D VAS = EQ-5D visual analog scale; MOS-HIV = Medical Outcome Study HIV Health Survey. P-value is significant at the 0.05 level.

^a^ Adjusted for all variables listed including collinearity between variables

## Discussion

The present study shows the high prevalence of pre-frailty/frailty (43.5%), and what is especially worrying, 39.8% were younger than 50 years.

In our first analysis, we observed that people with pre-frailty/frailty had more frequently hearing or visual impairment or falls, suggesting that it is necessary to incorporate geriatric principles for the management of theses patients. In this regards, Tassiopoulos et al. [20] observed that falls were more common among pre-frail/frail men. We also observed the same but the reasons are not well known. Erlandson et al. [21] notes that multiple potential factors (including tobacco use, comorbidities, medications) could be implicated in falls. Against what one would suppose, neither gait speed nor grip strength were associated with falls [20].

Continuing with this analysis, differences only versus frailty were observed after analyzing weight or height but not BMI. Similarly, other authors have observed that overweight and obesity are associated with a lower risk of frailty [22], potentially because being overweight or obese has been associated with an improved immune recovery and slower HIV disease progression, which may reduce the risk of frailty [23]. In contrast, other authors have observed that obesity is a risk factor for the development of frailty [24–26], potentially due to the low level of systemic inflammation related to this condition [24].

In relation to the Charlson score and VACS index, differences compared with frailty were also observed. Previously, different studies have also supported the use of the VACS index as a measure of frailty [19, 27, 28]. Although the VACS index does not routinely collect objective measurements of physical functions, this index is inversely correlated with quadriceps strength, grip strength, and 6-min walk distance [29]. In line with previous studies, our data also observed that higher VACS scores were associated with pre-frailty/frailty men [19, 30].

Comorbidities have a negative impact on frailty [5, 31]. Although not all of them have the same weight, the association of several of them can aggravate fragility. In our case, non-HIV related comorbidities but liver and bone comorbidities were associated with a higher risk of frailty, which has been described previously [32]. Although a physiological basis for the association could be determined, this observation should be interpreted with caution because these results could be the cause, or the consequence, of fragility. A similar phenomenon can also be reflected in polypharmacy for non-HIV therapies [32, 33].

In our study, creatinine levels reflected a worse situation in patients with pre-frailty/fragility, unlike ALT levels. Low serum ALT has been linked to frailty [34, 35], potentially because ALT is a surrogate marker for the hepatic ageing process [36]. It is interesting that the estimated glomerular filtration rate and albumin, both of which were lower in people with frailty, also allowed the differentiation of people with pre-frailty and frailty.

A surprising result is the absence of differences after analyzing multiple aspects related to HIV infection, including its different therapeutic options. Young et al. [37] did not find an association between frailty and CD4+ T cell counts, probably because only a small number of patients (21%) had a CD4+ T cell count <350 cells/mL. In our case, the average number of CD4+ cells was almost doubled. In our study, previous AIDS events were associated with a higher risk of pre-frailty/frailty in HIV-infected women. The reason could be that AIDS reflects a severe immunological damage that may contribute to pre-frailty/frailty [7, 38, 39].

Multivariable analysis (pre-frail/frail vs robust) also showed that CRP, which is regulated by IL-6, and especially menopause, were associated with frailty. In relation to CRP, this information could be useful in the surveillance of people with pre-frailty/frailty, although an important limitation is that it can be altered in response to numerous factors. Previously, Margolick et al. [40] observed that CRP was elevated in HIV frail men. Similar findings were reported by Erlandson et al. [41]. In our case, the reason why odds ratio was almost three times higher in women than in men is unknown. However, previous studies showed that high concentrations of CRP were more strongly predictive of incident frailty in women than in men [42]. Regarding menopause, there is ample evidence that menopause causes a loss of musculoskeletal tissue mass and quality, leading to poorer health and QoL [43]. These phenomena can be the loss of muscle mass at the onset of menopause [44] and the protective effect of estrogens in the muscle phenotype [45]. Indeed, these patients showed an increase in pro-inflammatory serum markers (IL1, IL6, TNF-alpha) [46]. Previously, in a well-defined group of postmenopausal HIV-infected compared with uninfected women, Young et al. [37] observed the occurrence of frailty earlier in HIV-infected women, although it was not statistically significant. More recently, in a study carried out in non-HIV infected patients, Verschoor et al [47] described in a cross-sectional analysis of the Canadian Longitudinal Study of Aging that age at menopause was inversely related to frailty. Unfortunately, we did not have data regarding the age of menopause occurrence or its reason. However, surgical menopause has not been associated with a greater risk for frailty than natural menopause [48].

Finally, people with frailty have a poorer QoL. Because QoL is closely related to functional capacity and other factors implicated in self-care and socialization, among others, it is necessary to address this problem, not only as a simple physical limitation. In our case, pre-frailty/frailty men had a lower EQ-5D, which have a negative impact on QoL. This finding has prognostic implications because in seronegative elderly people, the EQ-5D questionnaire had been demonstrated to be a useful predictor of mortality and hospitalization [49]. Similar findings have been described in HIV-infected adults, even after adjusting for CD4 and HIV VL [50]. Meanwhile, in pre-frailty/frailty women, only MOS-HIV PHS, showed significant differences.

The main limitation of this study is its cross-sectional design. Another limitation could be the small number of frail patients, which could limit our analysis. However, our results are consistent with previous studies [38]. It must be considered that frailty arises as a consequence of different chronic events and the accumulation of deficits; however it can be reversible. Another relevant aspect of our study was the inclusion of patients under 50 years of age, an age group that it is usually excluded in similar research [18, 27], in which frailty was observed. This finding implies that frailty is not closely related to age but to other circumstances such as an inflammatory state and the presence of comorbidities, among others.

In summary, factors associated with pre-frailty/frailty in HIV-infected patients differ by gender, which should be considered when establishing measures for prevention. The role of menopause in the risk of pre-frailty/frailty warrants further investigations.

## Supporting information

S1 File Spanish protocol. (PDF)

S2 File English protocol. (PDF)
