# Supplementary material for: Gender differences for frailty in HIV-infected patients on stable antiretroviral therapy and with an undetectable viral load
Source: PLoS One. 2019 May 9;14(5):e0215764. doi: 10.1371/journal.pone.0215764 (PMC6508723; doi:10.1371/journal.pone.0215764)
Supplement: S1 File — (PDF) [file pone.0215764.s001.pdf]

Número:……….

Demográficos:

- Fecha nacimiento: …………………………
- Fecha inclusión del paciente en el estudio: …………………………
- Edad autopercibida (en años): ………………..
- Sexo: Hombre □ Mujer □
- Raza/etnia: Caucásica □ Latinoamericana □ Subsahariana □
  Norte Africano □ Otros □
- Lugar de residencia: Urbano (Logroño, Calahorra, Arnedo, Haro) □ Rural □ [<10.000 hab.]
- Nivel cultural (auto-reportado): Sin estudios □ Primaria completa □
  Secundaria completa □ Universitario □
- Situación laboral (auto-reportado): Estudiante □ Trabajador activo □
  Desempleado □ Pensionista □
- Situación personal (auto-reportado): Vive sólo □ En familia □ Con amigos □
- Estatus marital (auto-reportado): Soltero/a □ Casado/a □ En pareja □
  Separado/a □ Viudo/a □

Estilo de vida (auto-reportado) y calidad de vida:

- Consumo de alcohol diario: Abstemio □ Leve <3/sem □ Moderado 3-13/sem □ Severo >14/sem □
- Tabaquismo: Nunca □ Exfumador [no fuma desde hace >6] □ Fumador □
- Actividad física recomendada por la OMS (en 18-64 años, ≥150 min/sem en una práctica aeróbica de intensidad moderada, o ≥75 min/sem de actividad física vigorosa, o bien una combinación equivalente de actividades moderadas y vigorosas): Si □ No □
- Consumo marihuana: Si □ No □
- Calidad de vida (MOS-VIH) (en español) https://eprovide.mapi-trust.org/instruments/medical-outcome-study-hiv-health-survey
- EQ-5D (en español) https://euroqol.org

Variables relacionadas con la presencia de comorbilidades:

- Indice comorbilidad Charlson (https://www.mdcalc.com/charlson-comorbidity-index-cci) e índice VACS (https://www.mdcalc.com/veterans-aging-cohort-study-vacs-index)
- Comorbilidades
  - cardiovascular (HTA, claudicación, ACV, insuficiencia cardiaca congestiva, angina de pecho, IAM) Si □ No □
  - digestivas (cirrosis hepática) Si □ No □
  - endocrinológicas (diabetes mellitus, obesidad, dislipemia, síndrome metabólico) Si □ No □
  - respiratorias (EPOC) Si □ No □
  - osteo-articulares (osteoporosis, fractura previa) Si □ No □
  - neuro-psiquiátricas (demencia, depresión, ansiedad, trastorno bipolar, esquizofrenia) Si □ No □
  - nefrológicos (insuficiencia renal crónica) Si □ No □

- Multimorbilidad (presencia de >2 comorbilidades) Si □ No □
- Polifarmacia (>5 fármacos no relacionados con el VIH) Si □ No □
- Número de fármacos para el VIH: …………

- Toma específica de algunos de estos tratamiento
  - AAS Si □ No □
  - Beta-bloqueantes Si □ No □
  - Diuréticos Si □ No □
  - IECA Si □ No □
  - ARA-II Si □ No □
  - Suplementos tiroideos Si □ No □
  - Estatinas Si □ No □
  - AINES Si □ No □
  - Antidepresivos Si □ No □
  - Anticolinérgicos Si □ No □

- Hospitalización el año previo por cualquier motivo: Si □ No □
- Autopercepción de su estado de salud: Excelente □ Muy buena □ Buena □ Regular □ Mala □
- Caídas por cualquier motivo el año previo Si □ No □
- Déficit neurosensorial (problemas de audición y/o que requieren audífono, y/o pacientes con dificultad para ver a pesar de llevar gafas): Si □ No □

- En las mujeres: - Embarazo previos Si □ No □
  - Número de embarazos ....
  - Menopausia (ausencia de regla >6 meses) Si □ No □
- A nivel familiar (familiares de primer grado): - Antecedente de IAM (en <60 años) Si □ No □
  - Hipertensión Si □ No □
  - Diabetes mellitus tipo 2 Si □ No □
  - Hipercolesterolemia Si □ No □

Relacionados con la infección por el VIH y otras coinfecciones:

- Fecha de diagnóstico VIH ....
- Vía de adquisición: Hetero □ HSH □ UDVP □ Otros □
- Fecha de inicio del TAR ....
- TAR actual: ....
- TAR previos empleados: NRTI □ NNRTI □ IPs □ INSTI □ Otros □
- Nadir de CD4:...............
- Evento sida previo: Si □ No □
- Lipoatrofia: Si □ No □
- Lipoacumulación: Si □ No □
- Coinfección VHB (HBsAg+) Si □ No □
- Coinfección VHC (ARN+): No □ Previa (curada) □ Activa □
- Adherencia al tratamiento (autoreportado): <85% □ 85-99% □ 100% □

Otros

- Velocidad de deambulación (4 m)..............
- Peso .... Talla ....
- Circunferencia de la cintura .... Circunferencia de la cadera ....

Analíticos

- RNA VIH <50 cop/mL Si □ No □
- RNA VIH <50 cop/mL desde have >1 año Si □ No □
- Linfocitos CD4
- Parámetros bioquímicos, hematológicos y de coagulación (ficha analítica)

Criterios de fragilidad

(Fried LP, et al. The Journals of Gerontology Series A, Biological sciences and medical sciences. 2001;56(3):M146-56).
