# Supplementary material for: Gender differences for frailty in HIV-infected patients on stable antiretroviral therapy and with an undetectable viral load
Source: PLoS One. 2019 May 9;14(5):e0215764. doi: 10.1371/journal.pone.0215764 (PMC6508723; doi:10.1371/journal.pone.0215764)
Supplement: S2 File — (PDF) [file pone.0215764.s002.pdf]

**Number:**..........

**Demographics**:

- Date of birthday: ...............................
- Date of inclusion in the study: ...............................
- Self-perceived age (years): ………………..
- Sex: Male □ Female □
- Race/ethnicity: Caucasian □ Latin American □ Sub-Sahairan □
  North African □ Other □
- Place of residence: Urban (Logroño, Calahorra, Arnedo, Haro) □ Rural □ [<10,000 inhab.]
- Cultural level (self-reported): No education □ Complete primary □ Full secondary □ University □
- Employment status (self-reported): Student □ Active worker □
  Unemployed □ Pensioner □
- Personal situation (self-reported): Live alone □ In family □ With friends □
- Marital status (self-reported): Single □ Married □ With a ouple □
  Divorced □ Widowed □

**Lifestyle (self-reported) and quality of life:**

• Daily alcohol consumption: Abster □ Mild <3 / week □ Moderate 3-13 / week □ Severe> 14 / week □

• Smoking: Never □ Ex-smoker [has not smoked for> 6] □ Smoker □

• Physical activity recommended by the WHO (in 18-64 years, ≥150 min/wk in an aerobic practice of moderate intensity, or ≥75 min / wk of vigorous physical activity, or an equivalent combination of moderate and vigorous activities): Yes □ No □

• Marijuana use: Yes □ No □

• Quality of life (MOS-HIV): https://eprovide.mapi-trust.org/instruments/medical-outcome-study-hiv-health-survey

• EQ-5D: https://euroqol.org

**Variables related to the presence of comorbidities**:

• Charlson comorbidity index (https://www.mdcalc.com/charlson-comorbidity-index-cci) and VACS index (https://www.mdcalc.com/veterans-aging-cohort-study-vacs-index)

• Comorbidities

- cardiovascular (HBP, claudication, stroke, congestive heart failure, angina pectoris, AMI) Yes □ No □
- digestive (liver cirrhosis) Yes □ No □
- endocrinological (diabetes mellitus, obesity, dyslipidemia, metabolic syndrome) Yes □ No □
- respiratory (COPD) Yes □ No □
- osteoarticular (osteoporosis, previous fracture) Yes □ No □
- neuro-psychiatric (dementia, depression, anxiety, bipolar disorder, schizophrenia) Yes □ No □
- nephrological (chronic renal failure) Yes □ No □

• Multimorbidity (presence of> 2 comorbidities) Yes □ No □

• Polypharmacy (> 5 drugs not related to HIV) Yes □ No □

• Number of drugs for HIV: ............

• Specific taking of some of these treatments

- AAS Yes □ No □
- Beta-blockers Yes □ No □

- Diuretics Yes □ No □
- ACEI Yes □ No □
- ARA-II Yes □ No □
- Thyroid supplements Yes □ No □
- Statins Yes □ No □
- NSAIDs Yes □ No □
- Antidepressants Yes □ No □
- Anticholinergic Yes □ No □

• Hospitalization the previous year for any reason: Yes □ No □
• Self-perception of your health status: Excellent □ Very good □ Good □ Fair □ Bad □
• Falls for any reason the previous year Yes □ No □
• Neurosensorial deficit (hearing problems and / or those that require a hearing aid, and / or patients with difficulty seeing despite wearing glasses): Yes □ No □
• In women:
- Previous pregnancy Yes □ No □
- Number of pregnancies .........
- Menopause (absence of period> 6 months) Yes □ No □

**At the family level (first degree relatives):**
- History of AMI (in <60 years) Yes □ No □
- Hypertension Yes □ No □
- Diabetes mellitus type 2 Yes □ No □
- Hypercholesterolemia Yes □ No □

**Related to HIV infection and other coinfections:**
• HIV diagnosis date ..................... ..
• Acquisition route: Heterosexual □ MSM □ UDVP □ Other □
• Start date of the ART .................. ..
• Current ART: ................................... ..
• Previous ART employed: NRTI □ NNRTI □ PIs □ INSTI □ Other □
• CD4 Nadir: ...............
• Previous AIDS event: Yes □ No □
• Lipoatrophy: Yes □ No □
• Lipoaccumulation: Yes □ No □
• HBV Coinfection (HBsAg +) Yes □ No □
• HCV Coinfection (RNA +): No □ Previous (Cured) □ Active □
• Adherence to ART treatment (self-reported): <85% □ 85-99% □ 100% □
• Others
  - Ambulation speed (4 m) ...............
  - Weight ………….
  - Size ……..…
  - Circumference of the waist .............
  - Hip circumference ...... .. ...

**Analytics parameters**

- HIV RNA <50 cop / mL Yes □ No □
- HIV RNA <50 cop / mL since have> 1 year Yes □ No □
- CD4 number
- Biochemical, haematological and coagulation parameters (analitical data)

**Fragility criteria (Fried Criteria):** criteria described by Fried et al. (Fried LP, et al. The Journals of Gerontology Series A, Biological sciences and medical sciences. 2001;56(3):M146-56).
